# Detection of Ebola Virus Antibodies in Fecal Samples of Great Apes in Gabon

**DOI:** 10.3390/v12121347

**Published:** 2020-11-24

**Authors:** Illich M. Mombo, Matthieu Fritz, Pierre Becquart, Florian Liegeois, Eric Elguero, Larson Boundenga, Telstar N. Mebaley, Franck Prugnolle, Gael D. Maganga, Eric M. Leroy

**Affiliations:** 1 Département de Virologie, Centre International de Recherches Médicales de Franceville, Franceville BP769, Gabon; boundenga@gmail.com (L.B.); telstarlunique@gmail.com (T.N.M.); gael_maganga@yahoo.fr (G.D.M.); 2 Institut de Recherche pour le Développement (IRD), Maladies Infectieuses et Vecteurs: Ecologie, Génétique, Evolution et Contrôle (MIVEGEC) (IRD 224—CNRS 5290—Université de Montpellier), BP 64501 Montpellier, France; matthieu.fritz@ird.fr (M.F.); pierre.becquart@ird.fr (P.B.); florian.liegeois@ird.fr (F.L.); eric.elguero@ird.fr (E.E.); franck.prugnolle@ird.fr (F.P.); 3 Institut National Supérieur d’Agronomie et de Biotechnologies (INSAB), Université des Sciences et Techniques de Masuku (USTM), Franceville BP913, Gabon

**Keywords:** great ape, Ebola, Africa, Luminex

## Abstract

Based on a large study conducted on wild great ape fecal samples collected in regions of Gabon where previous human outbreaks of Ebola virus disease have occurred between 1994 and 2002, we provide evidence for prevalence of *Zaire ebolavirus* (EBOV)-specific antibodies of 3.9% (immunoglobulin G (IgG)) and 3.5% (immunoglobulin M (IgM)) in chimpanzees and 8.8% (IgG) and 2.4% (IgM) in gorillas. Importantly, we observed a high local prevalence (31.2%) of anti-EBOV IgG antibodies in gorilla samples. This high local rate of positivity among wild great apes raises the question of a spatially and temporally localized increase in EBOV exposure risk and the role that can be played by these animals as sentinels of the virus’s spread or reemergence in a given area.

## 1. Introduction

Ebolavirus disease (EVD) is caused by one of the most feared and virulent pathogens currently threatening human populations [1]. It is characterized by hemorrhagic fever syndrome in both human and non-human primate (NHP) hosts following zoonotic infection with Ebolavirus. There are currently six species within the genus *Ebolavirus*: *Zaire ebolavirus* (EBOV), *Bundibugyo ebolavirus* (BDBV), *Sudan ebolavirus* (SUDV), *Tai Forest ebolavirus* (TAFV), *Reston ebolavirus* (RSTV), and a new species discovered in bats named *Bombali ebolavirus* (BOMV) [1]. Except for RSTV, all species are found in Africa. Three species, EBOV, BDBV, and SUDV, were responsible for the 28 human epidemics of EVD reported across Africa since the first recorded outbreak in 1976 [2,3]. Moreover, in 1994, TAFV was responsible for an outbreak devastating the chimpanzee population in Tai Forest, Ivory Coast. TAFV has only once been identified in a human, an ethologist contaminated during an autopsy of an infected chimpanzee [4,5]. By contrast, EVD arising from RSTV has only been observed in NHPs, and infections in humans appear asymptomatic [6,7]. The potential for infection in humans and NHPs by BOMV is still unknown under natural conditions, but experiment using pseudotype virus demonstrated that BOMV could enter human cells [8].

Outbreaks of EVD have caused deaths in chimpanzees, gorillas, and duikers [4,9], resulting in massive and rapid declines in great ape populations in Gabon and the Republic of the Congo [10,11]. Regarding human outbreaks, several epidemics appear to have started following viral transmission from wild animals, such as great apes [12]. Contact through the handling of infected chimpanzee and gorilla carcasses during butchering is the likely origin of human EBOV infections in Gabon and the Republic of the Congo and butchering an arboreal monkey is suspected to be at the origin of the index case in the recent Democratic Republic of the Congo (DRC) outbreak [9,13,14,15]. Whereas bats are considered a potential viral reservoir, their role in the natural cycle of EBOV is still unclear [16,17], as is the role of potential intermediate host species.

Analyses of blood or fecal samples in wild and captive NHPs, using diverse detection methods such as immunofluorescence assays, ELISA, and Western blots, report EBOV antibodies being present in up to 17.6% of samples tested [18,19,20,21]. These findings suggest that great apes are exposed to EBOV and that post-epidemic circulation may continue in some areas, as well as in areas where no outbreak has been reported. A better understanding of Ebola virus circulation in wildlife is needed to prevent future EVD outbreaks.

Here, we present results from molecular analyses of Ebolavirus and Marburg virus RNA as well as a multiplexed assay for detection of anti-EBOV immunoglobulin G (IgG) and immunoglobulin M (IgM) antibodies in great ape fecal samples collected at various locations in northeast Gabon over a four-year period.

## 2. Materials and Methods

### 2.1. Ethics Statement

Permission for noninvasive collection of great ape fecal samples was obtained from the National Center for Scientific and Technological Research (CENAREST), under authorization number AR0031/09 (11/01/2010). Serum samples from Ebolavirus disease survivors were collected during the Maybout outbreak in Gabon in 1996. Sample collection followed the regulation described in previous articles [13,22].

### 2.2. Sample Collection and RNA Isolation

**Figure 1:**
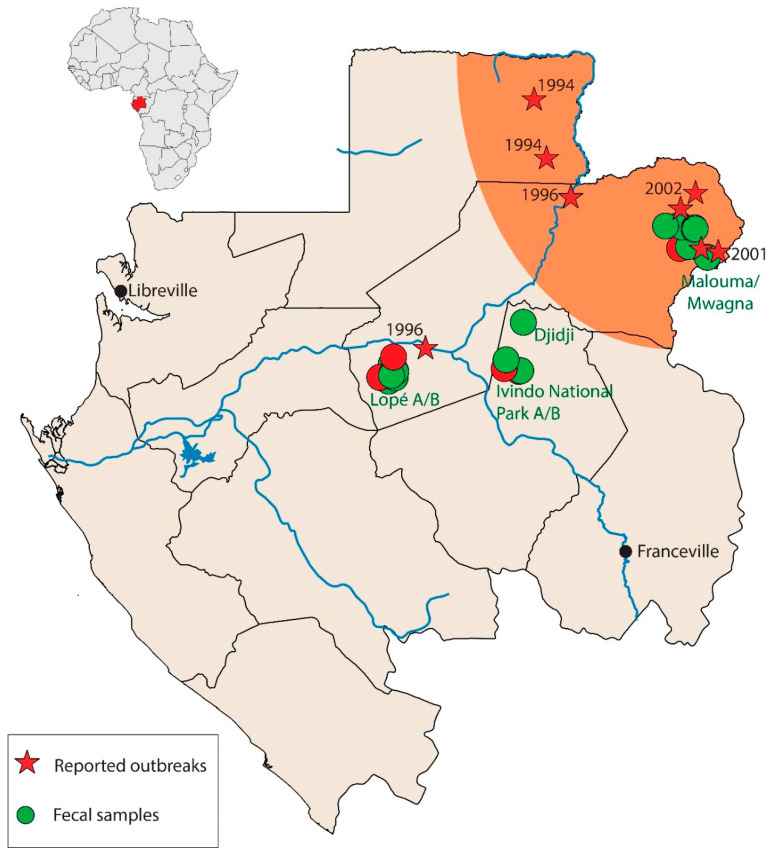
Sampling locations. The area of sharp great ape population decline is highlighted in orange. Green circles represent the sites where fecal samples were collected; red circles represent sites where positive fecal samples were collected. Red stars represent locations where human *Zaire ebolavirus* (EBOV) epidemics occurred.

A total of 444 great ape fecal samples (235 from gorillas and 209 from chimpanzees) were collected between 2009 and 2012, mainly in the Ogooué-Ivindo province, Northeast Gabon (Figure 1). These samples were used for the detection of Plasmodium species, enteric viruses, and simian immunodeficiency virus. Samples were collected as described previously [23,24]. Briefly, approximately 20 g of feces was preserved in a 20 mL solution of RNAlater (Ambion, Illkirch, France) until arrival in the laboratory, where it was then stored at −80 °C. Host species identification of each sample, determined previously, was based on observations in the field of feces morphology, vocalizations, night nests, locations where great apes were seen, and molecular methods [23,24]. Samples were classified into seven groups depending on the sampling site and date of collection (Table 1). Before extraction, RNAlater was removed by centrifugation, and fecal samples were pooled by up to 5 samples according to the site and the host species. The RNA was extracted from the pellet using the RNAqueous 4PCR RNA isolation kit (Thermo Fischer Scientific, Illkirch, France), following a modified version of the manufacturer’s procedures, and stored at −20 °C.

### 2.3. EBOV, SUDV, BDBV, and MARV RNA Detection

Filoviruses from the isolated RNA were first detected using specific quantitative real-time PCR (qPCR) and then with a nested reverse-transcription PCR (nRT-PCR). Reverse transcription in preparation for the qPCR analysis was performed using a high capacity cDNA reverse transcription kit (Applied Biosystems) and cDNA probed with primers specific for the detection of BDBV, SUDV, EBOV, and MARV (Table 2) using the TaqMan fast universal PCR master mix (Applied Biosystems, Illkirch, France). The nRT-PCR was performed using two pairs of degenerate primers targeting a 298 bp fragment of the L gene [9]. The first round was performed using the Superscript III one-step RT-PCR with Platinum Taq kit (Life Technologies, Illkirch, France) according to the manufacturer’s procedure. The reactions were run with a mix of 600 nM of each primer (Filo A and Filo B) with 5 µL of RNA in a 25 µL final volume. The second round was performed with the Platinum Taq DNA polymerase kit (Life Technologies), with reactions consisting of 600 nM of primers (Filo C and Filo D) and 2 µL of the first-round product in a final volume of 50 µL. Positive controls using synthetic RNA for each virus were processed at the same time.

### 2.4. Multiplexed Analysis of Anti-EBOV Antibodies in Great Ape Fecal Samples

#### 2.4.1. Antigens

Four commercially available recombinant proteins (GP, NP, and VP40) from *Zaire ebolavirus* (EBOV) were used as antigens in the immunoassay. The recombinant proteins used for this multiplexed assay are identical to those used in a previous study [27]. Briefly, GP was derived from strain Mayinga 1976 and Kissidougou-Makona 2014 (Sinobiologicals, Beijing, China), VP40 was derived from strain Kissidougou-Makona 2014 (Sinobiologicals), and NP was derived from Mayinga 1976 (MyBiosource, San Diego, CA, USA).

#### 2.4.2. Recombinant Antigen Coupling to Microsphere

Recombinant antigens were covalently coupled to a spectrally distinct microsphere (Magplex Microsphere, Luminex Corp., Austin, TX, USA). Briefly, 2 µg of each antigen was coupled to 1.25 × 10^6^ beads using a BioPlex amine coupling kit (Bio-Rad) according to the manufacturer’s procedure.

#### 2.4.3. Multiplexed Immunoassay

A microsphere-bead-based immunoassay of non-human primate fecal samples was tested for the presence of antibodies against the recombinant EBOV antigens. For each sample, the fecal/RNAlater mixture was first centrifuged (3900× *g* for 10 min), and the supernatant was then diluted in the assay buffer (phosphate-buffered saline (PBS)-1% Bovine Serum Albumin (BSA)-0.05% Tween 20) to a final dilution of 1:6, followed by incubation for 30 min at 56 °C. Inactivated samples (50 µL) were then tested in the Luminex assay, as previously described [18]. Briefly, 50 µL of each sample was mixed with coated beads (about 1250 of each type) and incubated in the dark overnight at 4 °C on an orbital shaker at 600 rpm. After three wash cycles with wash buffer (BioPlex wash buffer, Bio-Rad Laboratories, Marnes-la-Coquette, France) to remove unbound antibodies, 50 µL of 4 µg/mL antihuman biotin-labeled IgG or IgM (Jackson ImmunoResearch Europe Ltd., Cambridge, UK) in assay buffer was transferred to each well and incubated on an orbital shaker at 27 °C for 30 min at 700 rpm in the dark. After washing, the beads were incubated for 10 min at 27 °C at 700 rpm with 50 µL of Streptavidin-R-Phycoerythrin (SAPE) diluted to 4 µg/mL in assay buffer. After another washing, beads were resuspended in 100 µL of assay buffer, and measurements performed using a Magpix instrument (Luminex Corp., Austin, TX, USA). At least 100 events were read for each bead set, and binding events are displayed as median fluorescence intensities (MFIs). Each fecal sample was tested twice. In the absence of positive and negative fecal sample controls from great apes, the seropositivity cut-off value was calculated for each antigen as three standard deviations above the mean MFI of the total of samples. Although microsatellite identification was not carried out, we assumed that each sample represented a separate individual. Only 355/444 samples were evaluated due to quantity limitations after RNA investigation.

### 2.5. Statistical Analysis

We examined the effects of host species and sampling location on observed prevalences using binomial generalized linear models. All computations were performed with R software [28].

## 3. Results

### 3.1. No Viral RNA Detection

Neither Ebolavirus nor Marburg virus RNA was detected in the fecal samples of any chimpanzee or gorilla, whereas EBOV, BDBV, SUDV, and MARV RNA was detected in all positive controls.

### 3.2. Feasibility of Antibody Detection in a Small Fecal Sample Volumes

Several studies have previously demonstrated the possibility of antibody detection in RNAlater-preserved fecal samples from great apes [18,21,29], including a recent study using a multiplexing assay [18]. Compared to these previous studies, we had limited sample volume at our disposal and therefore needed to adapt existing protocols. We first assessed the ability to detect RNAlater-conserved antibodies without the PBS dialysis step. Sera from an EBOV survivor was spiked directly in pure RNAlater at different concentrations, conserved for three weeks at room temperature, and then placed at −80 °C. These samples were then analyzed as described in the Section 2. We found that the assay sensitivity was similar to that observed for diluted sera in a buffer assay. This result confirms that antibodies could be detected in small volumes of RNAlater-conserved fecal material (data not shown). By eliminating the dialysis step, our protocol is easier and faster than those described previously and can be used to analyze old libraries where only small volumes are available. Lastly, because antibody detection in feces is less sensitive than analysis of plasma, we considered a sample as positive with at least one significant antigen detection, a criterion adopted in the previous multiplex study [18].

### 3.3. Prevalence of IgG and IgM Antibodies Against EBOV in Fecal Samples of Gorillas and Chimpanzees

Of the 444 fecal samples, 355 (230 chimpanzee and 125 gorilla) were tested for anti-EBOV IgG and IgM antibodies using the multiplex assay.

For IgG, 20 apes were positive (5.6%), including nine chimpanzees (3.9%) and 11 gorillas (8.8%). From theses samples, six (30%) bound to more than one EBOV antigen, one with GP and NP, three with GP and VP40, and two with NP and VP40. Among the positive chimpanzee samples, four were collected in Lopé National Park (LNP): 3 of 43 samples collected between October 2009 and February 2010 in Lopé A, and 1 of 79 collected in 2012 in Lopé B (Figure 1 and Table 1). The remaining five positives were found among the 90 samples collected in Malouma in 2012. For gorillas, 10 of the positive samples were found among 32 fecal samples collected in the Ivindo National Park (INP) B in 2012. The remaining positive was found among 26 samples collected in INP A in 2010 (Figure 1 and Table 1).

For IgM, 11 apes were positive (3.1%), including eight chimpanzees (3.5%) and three gorillas (2.4%). From these samples, only one bound both NP and VP40 antigen from EBOV. The eight positive chimpanzee samples were all collected in the LNP (Lopé A and B): 3 of 43 samples between the end of 2009 and 2010, and 5 of the 79 collected in 2012. The three positive gorilla samples were collected in the INP B in 2012. Remarkably, the vast majority of positive fecal samples of Gorilla gorilla gorilla (ten of eleven for IgG and all of the samples positive for IgM) were collected during the same mission to the INP B in April 2012. This observed high prevalence of 31.2% in INP B in 2012 (10 of 32 samples positive for IgG) was significantly greater than for other groups (*p*-value = 0.00009; Table 1). All three gorilla samples positive for anti-EBOV IgM antibodies (9.4%) at INP B were also positive for anti-EBOV IgG antibodies.

## 4. Discussion

This study reports a large survey of wild great ape fecal samples for antibodies against EBOV. The samples were collected in regions of Gabon, where previous human outbreaks of EVD caused by EBOV had occurred between 1994 and 2002 [13,30]. We observed an immunoprevalence of anti-EBOV antibodies of 3.9% (IgG) and 3.5% (IgM) in chimpanzees and 8.8% (IgG) and 2.4% (IgM) in gorillas. There was tremendous spatial and temporal variation in observed immunoprevalence. Importantly, we observed a significantly higher prevalence (31.2%) of anti-EBOV IgG antibodies in gorilla samples collected in INP B in 2012. This high prevalence is likely an underestimate as antibody detection in fecal samples is much less sensitive than analyses of blood samples.

Our results are largely consistent with previous studies. Immunoprevalence of anti-EBOV IgG in blood samples of great apes born in nature in Gabon but subsequently reared in captivity was 4.13%, with the same study finding a slightly higher prevalence in Cameroon (17.6%) and the DRC (14.3%) [19]. A 2018 Nigerian study reported a seroprevalence of 2.1% in blood samples from monkeys [20].

Two recent studies sought to detect anti-EBOV antibodies in fecal samples of great apes. Using Western blot analysis, Reed et al. found a prevalence of 10% among fecal samples collected in a region affected by Ebolavirus outbreaks in the Republic of the Congo, close to our sampling locations in Malouma and Mwagna [21]. Second, a multiplex analysis by Ayouba et al. that failed to detect anti-EBOV antibodies in ape fecal samples reported that up to 2.6% of monkey blood samples were positive, depending on the criteria chosen [18].

The high prevalence observed in 2012 in INP B is striking compared to those reported in the other studies mentioned above. One caveat is that in the absence of microsatellite identification of individuals, our observed high prevalence could have resulted from the resampling of the same individuals. Previous studies report resampling rates ranging from 1.716–4.5 [29,31].

Even in the absence of appropriate positive and negative controls, assay specificity is supported by the observed clustering of positive samples. A low specificity would result in a more homogeneous distribution of positive samples over all sampling zones. Further support for the assay’s specificity comes from our observation of samples binding with more than one antigen from EBOV, in line with the observations of Ayouba et al. with paired feces and plasma samples from EBOV survivors [18].

Due to the small sample size and the absence of EBOV RNA detection, we cannot prove there was active circulation of the virus in this large area at the time of sampling. However, the much greater prevalence in animals from INP B, together with the detection of IgM antibodies, suggests that gorillas had been exposed to EBOV shortly before sampling. Because gorillas are sedentary and territorial, we could be observing local circulation of the virus potentially within the same social group. However, such localized high prevalence would be surprising and does not fit the entire northeast region’s epidemiological profile, which has been homogeneously affected by Ebola epidemics with active virus circulation between 1994 and 2002. A spatially and temporally localized increase in EBOV exposure risk could explain the relatively high prevalence. One possible risk factor is an increase in the gorilla population density, about ten years after the Ebolavirus caused a massive decline in the gorilla and chimpanzee populations in Northern and Eastern Gabon [11,14,32]. Secondly, an increase in EBOV prevalence or viral load in the reservoir population could also affect exposure risk. Finally, ecological factors could change patterns of interaction between gorillas and the viral reservoir. For example, an unusually high fruiting rate could increase exposure risk by bringing potential reservoirs, such as bats, together with gorillas [16,33]. Some low-level exposure sources, such as fruit contaminated by the feces, urine, or saliva from bats, could result in abortive, asymptomatic, or mild-symptomatic infections [16,34].

In conclusion, combined with previous studies’ results, our analysis confirms a low global prevalence of central African NHPs positive for EBOV antibodies and suggests possible high local circulation of the virus. As EBOV infection is highly lethal in great apes, the number of IgG-positive individuals is expected to be low, meaning our prevalence estimates likely underestimate exposure in these populations. While great apes are not the reservoir of the Ebolavirus, it is essential to continue to explore and monitor EBOV circulation in these species. Their high susceptibility to infection, combined with their close evolutionary relationship with humans, places them in the role of sentinels of the virus’s spread or reemergence in a given area. Monitoring of great ape populations by visual surveillance, fecal, or nest counting in areas considered at risk [35], combined with analysis of fecal samples such as those reported here, may be vital in preventing future EVD outbreaks in nearby human populations.

## Figures and Tables

**Table 1:** Prevalence of immunoglobulin G (IgG) and immunoglobulin M (IgM) EBOV antibodies in fecal samples of apes by sampling zone in Gabon between 2009 and 2012.

Zone	Date Collected	Species	No. Tested	IgG Pos/Total Tested	IgM Pos/Total Tested
Lopé A	October 2009–February 2010	*Gorilla gorilla gorilla*	34	0/34 (0.0; 0.0–10.3)	0/34 (0.0; 0.0–10.3)
		*Pan troglodytes troglodytes*	43	3/43 (7; 1.4–19.0)	3/43 (7; 1.4–19.0)
Lopé B	April–November 2012	*Gorilla gorilla gorilla*	22	0/22 (0.0; 0.0–15.4)	0/22 (0.0; 0.0–15.4)
		*Pan troglodytes troglodytes*	79	1/79 (1.3; 0.0–6.8)	5/79 (6.4; 2.0–14.1)
Mwagna	July 2011	*Gorilla gorilla gorilla*	0	0/0	0/0
		*Pan troglodytes troglodytes*	5	0/5	0/5
Malouma	January–September 2012	*Gorilla gorilla gorilla*	10	0/10 (0.0; 0.0–30.8)	0/10 (0.0; 30.8)
		*Pan troglodytes troglodytes*	90	5/90 (5.5; 1.8–12.5)	0/90 (0.0; 0.0–4.0)
INP B	April 2012	*Gorilla gorilla gorilla*	32	10/32 (31.2; 16.1–50.0)	3/32 (9.4; 2.0–25.0)
		*Pan troglodytes troglodytes*	1	0/1	0/1
Djidi	June 2011	*Gorilla gorilla gorilla*	1	0/1	0/1
		*Pan troglodytes troglodytes*	4	0/4	0/4
INP A	July–September 2010	*Gorilla gorilla gorilla*	26	1/26 (3.8; 0.0–19.6)	0/26 (0.0; 0.0–13.2)
		*Pan troglodytes troglodytes*	8	0/8	0/8

Note: Sampling zones are shown in Figure 1. Data are presented as number positive (%; 95% confidence interval) unless otherwise indicated. Percentages were not calculated when number of samples tested was <10.

**Table 2:** Primers and probes used for the detection of the filoviruses.

Virus	Primers and Probes	Sequences 5′ to 3′	References
EBOV	EBOZNPCONF1862	AGCTACGGCGAATACCAGAGTT	
	EBOZNPCONR1943	CGTCCTCGTCTAGATCGAATAGG	
	EBOZNPCONP1885	6 FAM-CTCGGAAAACGGCATGAATGCACC-BHQ1	
SUDV	Soudan-F	GCCATGGITTCAGGTTTGAG	[25]
	Soudan-R	GGTIACATTGGGCAACAATTCA	
	Soudan-P	6-FAM-ACGGTGCACATTCTCCTTTTCTCGGA-BHQ1	
BDBV	Uganda-F	GAGAAAAGGCCTGTCTGGAGAA	
	Uganda-R	TCGGGTATTGAATCAGACCTTGTT	
	Uganda-P	6 FAM-TTCAACGACAAATCCAAGTGCACGCA-BHQ1	
MARV	MBGCONTAQMF1	GGACCACTGCTGGCCATATC	[26]
	MBGCONTAQMR3-1	GAGAACATITCGGCAGGAAG	
	MBG 5313	6 FAM-CCTAAACAGGCTTGTCTTCTCTGGGACTT-BHQ1	
	MBG 5313- Prb RAV	6 FAM-ATCCTGAATAAGCTCGTCTTCTCTGGGACTT-BHQ1	
Panfilo	Filo A	TATMGRAATTTTTCYTTYTCATT	[9]
	Filo B	ATGTGGTGGGYTATAAWARTCACTRACAT	
	Filo C	GCWAAAGCMTTYCCWAGYAAYATGATGG	
	Filo D	ATAAWARTCACTRACATGCATATAACA	
